# Identification and Functional Characterization of the *SaMYB113* Gene in *Solanum aculeatissimum*

**DOI:** 10.3390/plants13111570

**Published:** 2024-06-06

**Authors:** Songheng Yi, Qihang Cai, Yanbo Yang, Hongquan Shen, Zhenghai Sun, Liping Li

**Affiliations:** 1 College of Landscape and Horticulture, Southwest Forestry University, Kunming 650224, China; 18314130085@163.com (S.Y.); caiqihang98@163.com (Q.C.); 15758384066@163.com (H.S.); 2 College of Geography and Ecotourism, Southwest Forestry University, Kunming 650224, China; yangyanbo_bio@163.com; 3 College of Wetland, Southwest Forestry University, Kunming 650224, China

**Keywords:** *Solanum aculeatissimum*, *SaMYB113*, anthocyanin biosynthesis

## Abstract

The MYB transcription factors (TFs) have substantial functions in anthocyanin synthesis as well as being widely associated with plant responses to various adversities. In the present investigation, we found an unreported MYB TF from *Solanum aculeatissimum* (a wild relative of eggplant) and named it *SaMYB113* in reference to its homologous gene. Bioinformatics analysis demonstrated that the open reading frame of *SaMYB113* was 825 bp in length, encoding 275 amino acids, with a typical R2R3-MYB gene structure, and predicted subcellular localization in the nucleus. Analysis of the tissue-specific expression pattern through qRT-PCR showed that the *SaMYB113* was expressed at a high level in young stems as well as leaves of *S. aculeatissimum*. Transgenic *Arabidopsis* and tobacco plants overexpressing *SaMYB113* pertinent to the control of the 35S promoter exhibited a distinct purple color trait, suggesting a significant change in their anthocyanin content. Furthermore, we obtained three tobacco transgenic lines with significant differences in anthocyanin accumulation and analyzed the differences in anthocyanin content by LC-MS/MS. The findings demonstrated that overexpression of *SaMYB113* caused tobacco to have considerably raised levels of several anthocyanin components, with the most significant increases in delphinidin-like anthocyanins and cyanidin-like anthocyanins. The qRT-PCR findings revealed significant differences in the expression levels of structural genes for anthocyanin synthesis among various transgenic lines. In summary, this study demonstrated that the *SaMYB113* gene has a substantial impact on anthocyanin synthesis, and overexpression of the *SaMYB113* gene leads to significant modifications to the expression levels of a variety of anthocyanin-synthesizing genes, which leads to complex changes in anthocyanin content and affects plant phenotypes. This present research offers the molecular foundation for the research of the mechanism of anthocyanin formation within plants, as well as providing some reference for the improvement of traits in solanum crops.

## 1. Introduction

Anthocyanins are flavonoid secondary metabolic substances that have a broad distribution within the flowers, leaves, and fruits of plants [1]. Anthocyanins are one of the most important components in the formation of the color of plant tissues, but also an important quality evaluation criterion in the production process of fruits and vegetables [2,3]. Currently, there are about 700 anthocyanins identified in plants, and they have a function in numerous plant physiological functions [4]. There are six major groups of typical anthocyanins: pelargonidin, cyanidin, delphinidin, peonidin, and petunidin, along with malvidin [5]. The anthocyanins’ formation is regulated by structural genes. Researchers have categorized these genes into early biosynthetic genes (EBGs) (*F3H*, *F3’H*, *CHS*, and *CHI*) and late biosynthesis genes (LBGs) (*DFR*, *LDOX*, *ANR*, and *UFGT*) based on their metabolic pathways [6]. The control of anthocyanins is also significantly impacted by transcription factors. The MBW complex, comprising three transcription factor families, MYB, bHLH, and WD40, can control anthocyanins through controlling the structural genes [7,8]. MYB TFs can regulate the early biosynthesis of anthocyanins by binding to EBGs [9]. During the later stages of anthocyanin biosynthesis, the MBW complex, on the other hand, achieves regulation of downstream accumulation of anthocyanins by modulating EBGs [10,11].

The MYB TF family constitutes one of the most numerous TF families in higher plants as well as playing a significant part in developing and growing plants, environmental response, and physiological metabolism, etc. [12]. Four subfamilies of the MYB TF family can be distinguished based on the quantity of tandem repetitions in the amino acids: 1R-MYB, R2R3-MYB, R1R2R3-MYB, and 4R-MYB [13], with R2R3-MYB being the most numerous subfamily of the MYB family [14,15]. According to earlier research, R2R3-MYB is vital for plant growth and development [16]. Among multiple MYB TFs, the R2R3-MYB TF is the primary regulator of anthocyanin synthesis [14]. The regulation of anthocyanin synthesis by R2R3-MYB transcription factors has been reported in various plants such as eggplant [17], Chinese bayberry [18], and tomato [19]. The molecular mechanism of anthocyanin regulation in model plants including *Arabidopsis* and tobacco has already been comprehensively studied [13,20]. *MYB113* is a member of the R2R3-MYB transcription factors and serves a regulatory function within anthocyanin synthesis in various plants. In eucalyptus, *MYB113* is regulated by phytocytomine, thereby influencing anthocyanin synthesis [21]. In eggplant, *MYB113* interacts with *CBF* genes to control the expression of structural anthocyanin synthesis genes *SmCHS* along with *SmDFR* [22,23].

*S. aculeatissimum* is a wild relative of eggplant in the Solanaceae family [24]. *S. aculeatissimum* is highly resistant and has significant medicinal value, making it a valuable germplasm resource for enhancing eggplant traits [25]. *S. aculeatissimum* is also commonly used as an eggplant rootstock to improve eggplant resistance to diseases and abiotic stresses [26,27]. Currently, most of the research on the molecular mechanisms within *S. aculeatissimum* focuses on the medicinal constituents [28,29]. Anthocyanin has a special value in plant breeding work as a crucial component for assessing the quality of fruits and vegetables as well as for participating in the response to plant adversity. The *MYB113* gene was identified from *S. aculeatissimum*, and its function was comprehensively analyzed through a gene overexpression assay and LC-MS/MS. This study serves as a reference for comprehending the function of MYB TFs in anthocyanin synthesis and as a genetic resource for enhancing eggplant quality.

## 2. Results

### 2.1. Isolation and Characterization of SaMYB113

An R2R3-MYB gene was isolated from the *S. aculeatissimum* genome and named *SaMYB113* (GenBANK: PP057707) according to its homologue. The open reading frame (ORF) was 825 bp in length, encoding 275 amino acids with a predicted molecular weight of 31.65 kDa. The theoretical pI value of the SaMYB113 protein was 8.97, which is greater than 7, indicating that it is a basic protein. The instability index value was 42.14, which is higher than 40, indicating that it is an unsteady protein. The aliphatic index was 77.23, and the GRAVY value was −0.712, which is less than 0, indicating that *SaMYB113* is a hydrophilic protein. Subcellular predictions were confined to the nucleus. This result corresponds with the characterization of the *SaMYB113* gene as a transcription factor.

### 2.2. Phylogenetic Relationship of SaMYB113 Protein

**Figure 1:**
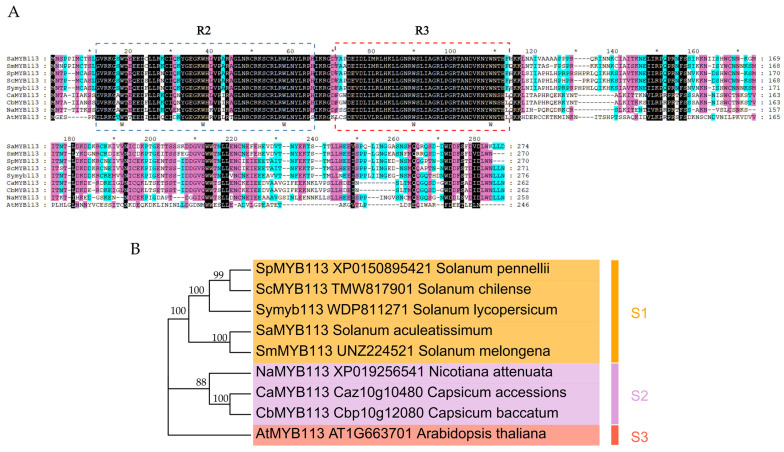
Alignment and phylogenetic relationship of SaMYB113 with various species of MYB TF proteins. (**A**) Amino acid sequence alignment of SaMYB113 with other species of MYB TF proteins. Blue and red boxes represent conserved structural domains. (**B**) Phylogenetic tree analysis of SaMYB113 as well as various species of MYB TF proteins.

To examine the evolutionary relationship between the SaMYB113 protein and MYB proteins in different plants, SaMYB113 as well as its homologous proteins were evaluated by multiple sequence alignment and phylogenetic study. Multiple sequence alignment showed that SaMYB113 has conserved structural domains (W-X19-W-X19-W and F/I-X18-W-X18-W) unique to R2R3-MYB (Figure 1A) [30]. The MYB113 proteins in different species can be categorized into three groups in terms of phylogenetic relationships (Figure 1B). With four species of Solanum including *S. aculeatissimum* in the S1 group, three species of solanaceae in the S2 group, and *Arabidopsis* as the only species in subgroup S3. It can be seen that the SaMYB113 protein is distantly phylogenetically related to MYB113 in Arabidopsis, and the MYB113 protein is highly conserved in solanum, suggesting that it is crucial to the growth and development of solanum. The close phylogenetic relationship between SaMYB113 and eggplant MYB113 proteins in solanum suggests a similar phylogeny of MYB113 proteins in *S. aculeatissimum* and eggplant.

### 2.3. The Expression Patterns of SaMYB113

**Figure 2:**
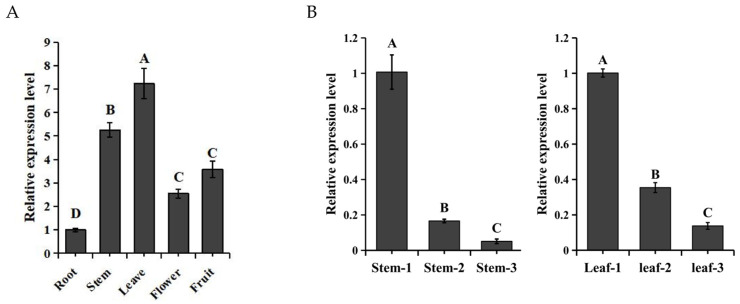
The expression patterns of *SaMYB113*. (**A**) The expression patterns of *SaMYB113* in a variety of tissues. (**B**) Expression pattern analysis of *SaMYB113* in stems as well as leaves at various growth stages. The relative expression levels are displayed on the y-axis, while the various samples are displayed on the x-axis. Significant variations between samples are displayed through different capital letters, as carried out through one-way ANOVA and Tukey’s study (*p* < 0.01). Error bars represent the standard deviation of technical repetitions.

For the purpose of understanding the regulatory ability of *SaMYB113* on different tissues, the *SaMYB113* expression levels within *S. aculeatissimum* roots, stems, leaves, and flowers, along with fruits were examined through qRT-PCR. The outcomes showed that the relative degrees of expression of the *SaMYB113* gene were significantly different within different tissues (Figure 2A). The lowest expression levels were found in roots; the relative expression levels within stems and leaves were noticeably greater compared to those in other tissues, demonstrating the fact that *SaMYB113* has a potentially significant impact on how leaves and stems grow and develop. In order to learn more about the potential regulatory mechanism of the *SaMYB113* gene within stems as well as leaves, the expression level of the *SaMYB113* gene was analyzed in the stem and leaves of young, growing, and mature tissues. The findings demonstrated that the expression level of the *SaMYB113* gene was notably greater in young tissues than within mature tissues (Figure 2B).

**Figure 3:**
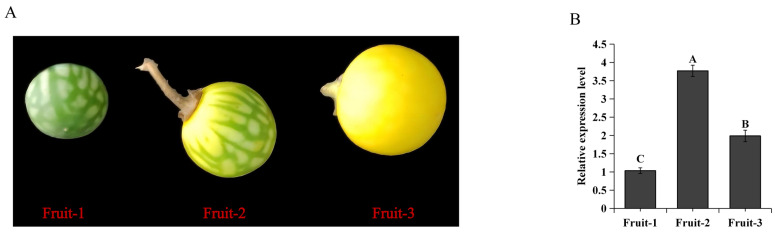

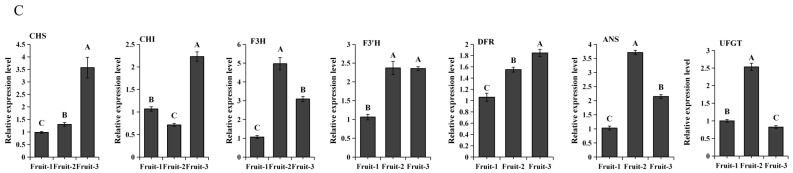
Analysis of gene expression levels during fruit color formation in *S. aculeatissimum*. (**A**) Fruits of *S. aculeatissimum* at different stages of growth, Fruit-1, 2, and 3 represent 10, 20, and 40 d after the end of flowering, respectively. In Fruit-1, no yellow color appeared, in Fruit-2, yellow color began to appear, and in Fruit-3, all fruits were yellow. (**B**) Expression levels of the *SaMYB113* gene in *S. aculeatissimum* fruits at different growth stages, with the y-axis representing the relative expression levels of the gene and the x-axis representing different growth stages. (**C**) Expression levels of structural genes for anthocyanin synthesis in *S. aculeatissimum* fruits at different growth stages, with the x-axis representing different growth stages and the y-axis representing relative expression levels. The significance of differences between different samples is shown by different capital letters, and the error bars represent the standard deviation of technical replicates.

In order to understand the potential regulatory mechanism of the *SaMYB113* gene in the process of *S. aculeatissimum* fruit color formation, the expression levels of the *SaMYB113* gene and anthocyanin synthesis genes in *S. aculeatissimum* fruits at different growth stages (Figure 3A) were analyzed. The results showed that *SaMYB113* gene expression levels differed significantly during the course of different fruit growth stages, with the highest relative expression levels in the Fruit-2 stage (Figure 3B). Analysis of the expression levels of structural genes for anthocyanin synthesis in *S. aculeatissimum* fruits at different growth stages showed that the structural genes for anthocyanin synthesis exhibited different expression patterns at different growth stages of the fruits (Figure 3C). *CHS* and *DFR* had similar expression patterns, with lower expression at the beginning of fruit growth, and increased gene expression levels with fruit growth; *F3H*, *ANS*, and *UFGT* had similar expression patterns, with significantly higher expression levels at the beginning of yellow coloration of the fruit; and *CHI* and *F3’H* had higher expression levels in all-yellow fruits. It can be seen that the *SaMYB113* gene has a similar expression pattern to *F3H*, *ANS*, and *UFGT* in fruits at different stages of growth and development, and these results suggest that the expression of the *SaMYB113* gene may regulate the structural genes for anthocyanin synthesis, thereby affecting the synthesis and accumulation of anthocyanins in *S. aculeatissimum*.

### 2.4. Overexpression of SaMYB113 Alters the Color of Arabidopsis Plants

**Figure 4:**
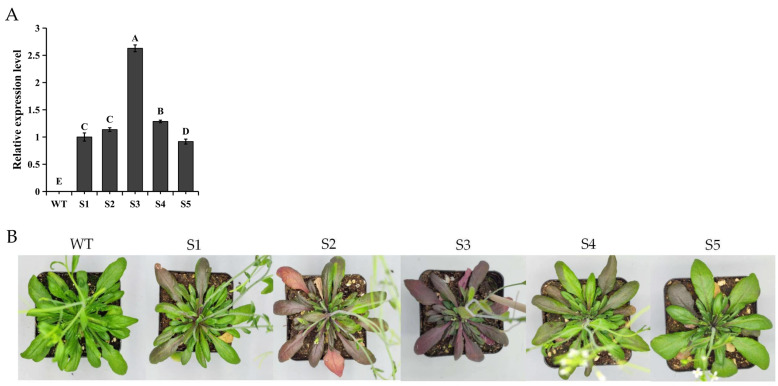
Overexpression of *SaMYB113* alters the color of *Arabidopsis* plants. (**A**) qRT-PCR detection of transgenic *Arabidopsis.* The relative expression levels are shown on the y-axis, and the various lines are shown on the x-axis. Based on a one-way ANOVA and Tukey’s test (*p* < 0.01), various capital letters among specimens suggest noteworthy distinctions. Error bars represent the standard deviation of technical repetitions. (**B**) *Arabidopsis* phenotypes of different *SaMYB113* overexpression transgenic lines. WT: wild type; S1, S2, S3, S4, S5: *SaMYB113* overexpression transgenic lines.

*SaMYB113* was introduced into Arabidopsis, and five T2 generation *Arabidopsis* transgenic lines (S1, S2, S3, S4, and S5) were examined by qRT-PCR using wild-type Arabidopsis (WT) as a control group (Figure 4A). The transgenic lines differed somewhat in expression levels, and cultivation of these lines was continued to obtain T3 generation purebred transgenic plants. By observing the morphology of the T3 generation, it was easy to find that the transgenic *Arabidopsis* leaf color produced significant changes compared with the wild type, and there was also a significant variation in leaf color among the different *SaMYB113*-expressing transgenic lines (Figure 4B). Specifically, with the rise in *SaMYB113* expression, the purple area of leaves increased and the color deepened, in regards to which the relative expression of the S3 gene line was greater compared to the other transgenic lines, as the plants showed a dark purple color.

### 2.5. Overexpression of SaMYB113 Alters the Color of Tobacco Plants

**Figure 5:**
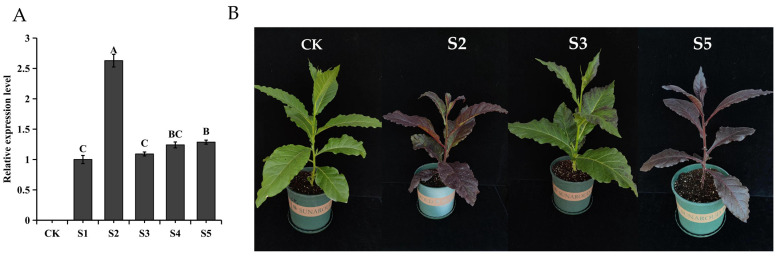
Overexpression of *SaMYB113* alters the color of tobacco plants. (**A**) Transgenic tobacco qRT-PCR validation: the y-axis shows the relative expression levels; the x-axis shows the various transgenic lines. Based on one-way ANOVA and Tukey’s test (*p* < 0.01), various capital letters among specimens suggest substantial distinctions. Error bars represent the standard deviation of technical repetitions. (**B**) Tobacco phenotypes of different overexpression *SaMYB113* transgenic lines. CK: control subjects (wild type); S2, S3, S5: *SaMYB113* overexpression transgenic lines.

The *SaMYB113* gene was overexpressed in tobacco, and six gene lines were obtained. The relative expression levels of the *SaMYB113* gene were quantified through qRT-PCR using wild-type tobacco as a control (Figure 5A). The outcomes showed that the *SaMYB113* gene was successfully integrated into the tobacco genome. S2, S3, and S5, which had significant differences in expression levels, were selected for cultivation, and significant differences in plant phenotypes were found among these three transgenic lines. Compared to the CK, the plants of all three transgenic lines had purple phenotypes in the leaves (Figure 5B). Specifically, the S3 transgenic line had the lowest leaf purple levels; the S2 transgenic line had intermediate leaf purple levels, and the S5 transgenic had the highest leaf purple levels. Notably, the expression level of the *SaMYB113* gene was considerably greater in the S2 gene line in contrast to other transgenic lines, but the level of purple color in its leaves was less than that in the S5 gene line, a phenomenon that may be due to the difference in anthocyanin composition. The differences in anthocyanin accumulation in these transgenic lines may be due to differences in the location and copy number of the SaMYB113 gene present in their transformation process.

### 2.6. Differential Analysis of Anthocyanins in Tobacco from Different Transgenic Lines

**Figure 6:**
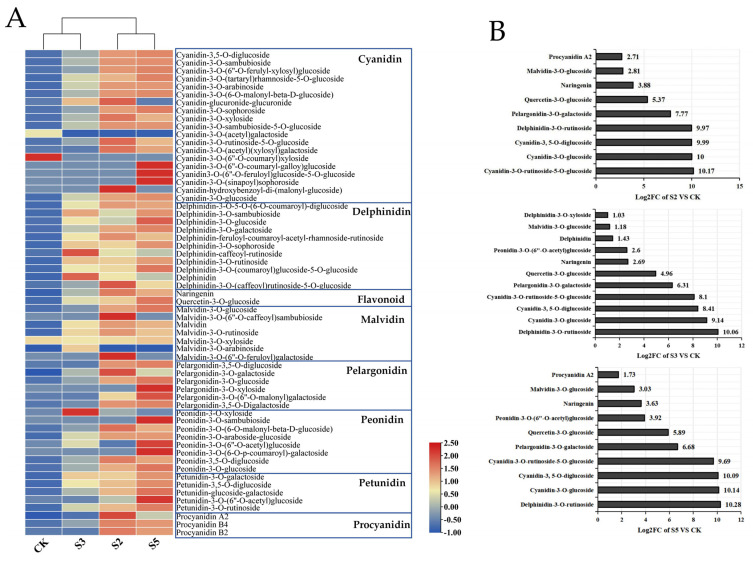
Differential analysis of anthocyanins in tobacco from different transgenic lines. (**A**) Heat map of anthocyanin content in different transgenic lines: blue boxes represent different anthocyanin species. (**B**) Anthocyanin compositions that were significantly different from the control in each genetic lineage: y-axis represents the names of different anthocyanins; x-axis represents the log2 (sample/control) value of anthocyanin content.

To examine the control mechanism of *SaMYB113* in anthocyanin synthesis, we quantitatively analyzed 61 anthocyanins from eight major classes (cyanidin, delphinidin, flavonoid, malvidin, pelargonidin, peonidin, and petunidin, as well as procyanidin) in various tobacco transgenic lines (Figure 6A). The results showed that anthocyanins increased to some extent in all three transgenic lines in contrast to the wild-type control. There was a substantial distinction in the content of 52 anthocyanins within the S2 gene line compared to the control, 47 anthocyanins within the S3 gene line compared to the control, and 54 of the anthocyanins in the S5 gene line compared to the control. Cluster analysis of anthocyanin content revealed that the S2 gene line clustered with the S5 gene line, and the CK clustered with the S3 gene line, which may be due to the significantly lower expression of the *SaMYB113* gene in the S3 gene line than within the S2 and S5 transgenic lines. Notably, we found a considerable decrease in cyanidin-3-O-(6″-O-coumaryl) xyloside content after overexpression of the *SaMYB113* gene, suggesting that the effect of *SaMYB113* overexpression on the anthocyanin synthesis pathway is complex. Analysis of the anthocyanin species with the most significant differences between each genetic line and the control revealed that the most significantly up-regulated anthocyanins were similar in the S3 and S5 transgenic lines, with the three most significantly up-regulated anthocyanins being delphinidin-3-O-rutinoside, cyanidin-3-O-glucoside, and cyanidin-3,5-O-diglucoside (Figure 6B). The three most significantly up-regulated anthocyanins in the S2 genetic line were cyanidin-3-O-rutinoside-5-O-glucoside, cyanidin-3-O-glucoside, and cyanidin-3,5-O-diglucoside. It can be seen that the most significantly up-regulated anthocyanin species in the S2 gene line differed from the S3 and S5 transgenic lines, which could be brought on through variations in the expression levels of genes regulating anthocyanin synthesis.

### 2.7. Analysis of Differences in Expression Levels of Structural Genes for Anthocyanin Synthesis in Different Tobacco Transgenic Lines

**Figure 7:**
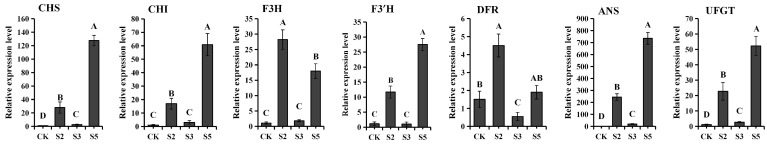
Analysis of differences in expression levels of structural genes for anthocyanin synthesis in different tobacco transgenic lines. The relative expression levels are displayed on the y-axis, while the various samples are displayed on the x-axis. Based on one-way ANOVA and Tukey’s test (*p* < 0.01), various capital letters within specimens suggest substantial differences. Error bars represent the standard deviation of technical repetitions.

To look into the underlying molecular processes of the variations in anthocyanin content among different tobacco transgenic lines, the expression levels of structural genes for anthocyanin synthesis within different transgenic lines were examined through qRT-PCR (Figure 7). The findings demonstrated that following overexpression of *SaMYB113*, the structural genes for anthocyanin synthesis within tobacco plants of different transgenic lines produced significant differences. The structural genes in the S3 gene line differed less than the control, with a substantial rise in the relative expression levels of *CHS*, *ANS*, and *UFGT*, and a substantial reduction in the relative expression level of *DFR*. The expression levels of structural genes for anthocyanin synthesis were notably higher in both the S2 and S5 transgenic lines than in the control and S3 transgenic lines. It is noteworthy that the relative expression levels of *CHS*, *CHI*, *F3’H*, *ANS*, and *UFGT* were substantially lower in S2 than in S5. By comparison, the relative expression levels of *F3H* and *DFR* were substantially greater in the S2 contrasted with the S5 gene line. Variations in the expression levels of these structural genes might be accountable for differences in anthocyanin accumulation between transgenic lines.

## 3. Discussion

Anthocyanin is an indigenous, water-soluble pigment, which is extensively observed in plants. Anthocyanins not only give colors to plant roots, stems, leaves, flowers, fruits, and seeds, as well as other organs, but also improve the ability of plants to reproduce offspring and resist the adverse environment. Therefore, the mechanism of anthocyanin formation in plants has always been a hot topic of research [31,32,33,34]. MYB TFs are the members of the biggest families of transcription factors in plants and are broadly associated with secondary metabolism, stress response, and signal transduction in plants, and play a significant part within the synthesis of anthocyanins in plants [35,36]. A significant number of studies have reported upon the regulation of anthocyanin synthesis by MYB genes, like *AtMYB75* along with *AtMYB90* in *Arabidopsis*; *FuMYB10* in tobacco; as well as *PpMYB7* in peach, all of which have been shown to be connected to the synthesis and control of anthocyanins [17,37,38,39]. *MYB113*, a member of the MYB gene family, has been shown to serve an essential function in anthocyanin synthesis within *Arabidopsis* [40], tomato [41] and eggplant [17]. We identified an R2R3-MYB gene from *S. aculeatissimum* based on sequencing data, which has not been documented in previous research. Protein sequence alignment demonstrated that it is extremely homologous to *MYB113* in a variety of plants and it is hypothesized to play significant function in anthocyanin synthesis. Bioinformatics analyses showed *SaMYB113* to be a basic, unstable, hydrophilic protein with a typical R2R3-MYB conserved structure, predicting the subcellular localization in the nucleus, and these results are consistent with those for *MYB113* in other plants [42,43]. Phylogenetic study demonstrated that the SaMYB113 protein has a similar phylogenetic connectivity to the MYB113 protein in eggplant and may have similar regulatory functions.

The expression of the MYB gene has significant spatiotemporal specificity [44,45]. In this study, *SaMYB113* was found to be expressed at high levels within young stem as well as leaf tissues and at low levels in mature tissues. This result suggests that this gene may play an important regulatory role in the early development of plant organs. In this study, we verified the gene function through overexpressing the *SaMYB113* gene in *Arabidopsis* as well as tobacco. The results showed that the plants overexpressing the *SaMYB113* gene showed different degrees of purple phenotype, implying that *SaMYB113* participated in the positive regulation of anthocyanin. Furthermore, plants from different transgenic lines exhibited different purple traits; this may be due to differences in gene copy number and gene binding sites among transgenic lines. Prior investigations have demonstrated that the MYB TFs can influence anthocyanin expression levels in plants by specifically activating structural genes for anthocyanin synthesis [46]. The expression of the structural genes for anthocyanin biosynthesis is greatly enhanced when MYB TFs bind to their promoters, activating the downstream network of anthocyanin biosynthesis [47]. For example, the *AcMYB75* gene in kiwifruit can bind to the promoter region of the *ANS* gene as well as regulate its expression level [48]; the *PsMYB12* gene in peonies interacts with bHLH and WD40 to stimulate the expression of *PsCHS* after the establishment of the MBW complex, resulting in pigmented plaques [49]; expression of *MdMYB10* in apple substantially raises the expression level of *MdDFR*, thereby increasing anthocyanin content [36]. In this study, we identified the expression levels of structural genes in tobacco from different transgenic lines overexpressing the *SaMYB113* gene through qRT-PCR, and the findings demonstrated that the expression levels of structural genes such as *CHS*, *CHI*, *F3H*, *F3’H*, *ANS*, and *UFGT* were up-regulated to multiple levels in different transgenic lines, indicating that the expression of *SaMYB113* can regulate the expression of structural genes for anthocyanin synthesis, thus regulating anthocyanin synthesis.

Anthocyanins have complex and diverse structures and species, and different anthocyanin species are regulated by different molecular mechanisms and have different functions [50]. In this study, the amounts of a number of anthocyanin components were significantly increased in transgenic tobacco plants overexpressing *SaMYB113*; alongside the most pronounced increases in cyanidin-like anthocyanins and delphinidin-like anthocyanins, the anthocyanin species that was highest differed among the transgenic lines. Thus, it can be seen that the *SaMYB113* gene can influence plant color formation through regulating anthocyanin biosynthesis structural genes and, thus, the synthesis of cyanidin and delphinidin-like anthocyanins. Due to the different transformation processes, the expression levels of the structural genes for anthocyanin synthesis differed in different transgenic lines of tobacco, which resulted in different levels of purple color in different transgenic lines.

## 4. Materials and Methods

### 4.1. Bioinformatics Analysis of SaMYB113

The *SaMYB113* sequence information was uploaded to NCBI database (https://www.ncbi.nlm.nih.gov/) (cDNA full-length sequence in the supplementary materials document) (accessed on 29 December 2023). Predictive analysis of protein physicochemical properties was performed using the Expasy website (https://web.expasy.org/protparam/) (accessed on 29 December 2023). Subcellular localization information was forecasted using the WoLF PSORT website (https://wolfpsort.hgc.jp/) (accessed on 29 December 2023). The amino acid sequences of *MYB113* within *Solanum melongena* (UNZ22452.1) [40], *Solanum pennellii* (XP_015089542.1), *Solanum chilense* (TMW81790.1), *Solanum lycopersicum* (WDP81127.1), *Capsicum accessions* (Caz10g10480) [51], *Capsicum baccatum* (Cbp10g12080), *Nicotiana attenuata* (XP_019256541) [24], and *Arabidopsis* (AT1G66370.1) [52] were obtained using the NCBI database (https://www.ncbi.nlm.nih.gov/) (accessed on 30 December 2023). Amino acid sequence alignment was undertaken employing MEGA11 v11.0.10 software and the results were visualized by Genedoc v2.7.0 software comparison. Utilizing the neighbor-joining (NJ) method with a 1000 bootstrap, phylogenetic trees were built.

### 4.2. S. aculeatissimum Material, Growth Condition, and Treatment

The wild-type *S. aculeatissimum* (ZHSa-7) used for expression pattern analysis was planted in the experimental greenhouse of Southwestern Forestry University (102.45° E, 25.04° N). Seeds were soaked in pure water for 24 h prior to sowing, after which they were germinated on moistened filter paper at 30 °C ambient temperature. Sprouted seeds were transferred to hole trays of 5.5 cm aperture for planting, and seedlings were transferred to 25 cm diameter planting pots with peat:vermiculite = 3:1 planting substrate after they had grown to 5 mature leaves. Expression patterns were analyzed when the plants were grown to the fruiting stage. Tissue expression pattern analysis was conducted by selecting roots, stems, leaves, flowers, and fruits. Stems and leaves from early growth stage (germination 5 days), middle growth stage (germination 15 days), and late growth stage (germination 25 days) were selected to analyze expression patterns during growth. The surface of the samples was rinsed with ddH_2_O, and the samples were immersed in liquid nitrogen, then kept at −80 °C.

### 4.3. Overexpression Vector Construction and qRT-PCR

RNA was obtained from the tissues by employing FastPure Plant Total RNA Isolation Kit (Vazyme Biotech Co, Ltd., Nanjing, China), and RNA was reverse-transcribed to cDNA by the All-In-One 5X RT MasterMix kit (Applied Biological Materials Inc., Shanghai, China). *SaMYB113* was cloned from the cDNA of *S. aculeatissimum* using the MegaFi™ Fidelity 2X PCR MasterMix (Applied Biological Materials Inc.). *SaMYB113* was ligated into the pCAMBIA 1302 vector (CaMV 35S promoter control) using the ClonExpress II One Step Cloning Kit (Vazyme Biotech Co., Ltd.). Agrobacterium tumefaciens (GV3101) was transformed using pCAMBIA1302-*SaMYB113*. The qRT-PCR primers were developed utilizing the primer-Blast function in the NCBI database (https://www.ncbi.nlm.nih.gov/) (accessed on 12 January 2023) (Table 1). the *TUA* gene was utilized as an internal reference gene for qRT-PCR, and the relative gene expression levels were calculated using 2^−ΔΔCt^ [53].

### 4.4. Transgenic Arabidopsis Acquisition

The agrobacterium was cultured to OD600 = 0.8–1.0 at 28 °C at 150 rpm using an LB medium including 50 mg/L kanamycin sulfate. Colombian wild-type *Arabidopsis* was selected for the experiment, and the floral dipping method was used for infestation [54]. Transgenic plants were screened using hygromycin (30 mg/L). Five transgenic lines were selected for quantitative fluorescence identification and morphological observation from culture to T3 generation.

### 4.5. Transgenic Tobacco Acquisition

The tobacco (*Nicotiana tabacum* cv k326) used for the study was grown in the experimental greenhouse at Southwest Forestry University and transgenic tobacco was obtained using the leaf disk method [55]. Mature leaves of tobacco free from pests and illnesses were chosen and sterilized in the aseptic table (75% ethanol immersion for 60 s, aseptic water rinsing 2 times, use of 0.1% HgCl_2_ immersion for 10 min, aseptic water rinsing 5 times), and the sterilized leaves were sliced into 2 cm^2^ squares for spare use. Agrobacterium was cultured at 28 °C and 150 rpm to OD600 = 0.8–1.0, harvested via centrifugation, and revived using a comparable volume of liquid MS medium. Leaves were soaked in resuspended agrobacterium solution for 10 min, after which the leaf surface was wiped dry of the agrobacterium liquid. They were cultured on solid MS medium containing 2.25 mg/L 6-BA and 0.3 mg/L NAA for 48 h. This was followed by screening culture in solid MS medium containing 2.25 mg/L 6-BA, 0.3 mg/L NAA, 20 mg/L hygromycin, and 400 mg/L cefotaxime. Shoots with hygromycin opposition were sliced and inoculated into solid MS medium including 20 mg/L hygromycin and 400 mg/L cefotaxime for rooting culture. When the seedlings developed a well-developed root system and the plants grew to a height of around 10 cm, they were taken away and cleaned with water for removing the root agar, and then transplanted to the greenhouse for cultivation.

### 4.6. Compositional Analysis of Tobacco Anthocyanins

Leaves of CK, S2, S3, and S5 tobacco plants free of pests and diseases were selected, and after rinsing the leaf surface using ddH_2_O, they were immersed in liquid nitrogen and rapidly frozen, after which they were maintained at −80 °C. The samples were delivered to Wuhan Maiwei Metabolic Biotechnology Co., Ltd. (Wuhan, China) for the determination of anthocyanin composition using the LC-MS/MS technique. Data acquisition was carried out through Ultra Performance Liquid Chromatography, UPLC, and Tandem Mass Spectrometry. The liquid chromatographic column was ACQUITY BEH C18 1.7 μm, 2.1 mm × 100 mm (Waters Technologies Co., Ltd., Milford, MA, USA). The mobile phase A was ultrapure water (containing 0.1% formic acid); phase B was methanol (containing 0.1% formic acid). The elution scheme was such that the percentage of phase B was 5% at 0 min, increased to 50% at 6 min, increased to 95% at 12 min and held for 2 min, and decreased to 5% at 14 min and equilibrated for 2 min. The UPLC chromatogram (TIC plot) can be seen in Figure S1. The flow rate of the liquid phase was 0.35 mL/min and the temperature in the column was 40 °C.

## 5. Conclusions

In this study, we reported on the *MYB113* gene in *S. aculeatissimum* for the first time, and performed a comprehensive bioinformatics analysis as well as an expression pattern analysis of *SaMYB113*. The function of the gene was verified by overexpression in *Arabidopsis* and tobacco, and the results showed that *SaMYB113* was regulating anthocyanin synthesis in transgenic plants. Differences in anthocyanin content in transgenic tobacco from different transgenic lines were detected by LC-MS/MS, and the expression levels of structural genes for anthocyanin synthesis were examined by qRT-PCR. The results indicate that *SaMYB113* can regulate the level of anthocyanin content in plants by affecting the expression level of structural genes for anthocyanin synthesis. This study provides a certain reference for understanding the regulatory mechanism of the *MYB113* gene on anthocyanin synthesis in plants, and also provides candidate genes for the breeding of improved anthocyanin traits in eggplant.

## Figures and Tables

**Table 1:** The primer sequences used in this research.

Gene Name	Forward Primer Sequence (5′ –> 3′)	Reverse Primer Sequence (5′ –> 3′)
*SaMYB113*	GCTTCTAGGCAACAGGTGGT	CTTTGACGAGGAGGAGGAGC
*CHS*	ACTCCGGATGGCTAAGGACT	ACCTATAATGACCGCGGCTG
*CHI*	CTCAATCACCTGTTGGGGCA	CACTTTGCTGCAGGGGAAAC
*F3H*	GGCCAGACAAACCAGATGGA	ATGCCTTGGTTAAGGCCTCC
*F3′H*	GTTGATCCACAGGCGGAAGA	AACCAATCGAGTGCCGGAAT
*DFR*	TGTGGCAACGCCTATGGATT	GGACATCGACAGTTCCAGCA
*ANS*	AGGACCTCAAGTACCGACGA	TTCCAGCAACCTTGACACGA
*UFGT*	CACTTCACAATCCAACACCTCA	CGTCCAATAGGGGTAGTGCC
*TUA*	GATGTCAATGCTGCTGTCGC	CCAGGCACTACTGTTGGAGG

## Data Availability

Data are contained within the article and Supplementary Materials.

## Supplementary Materials

The following supporting information can be downloaded at: https://www.mdpi.com/article/10.3390/plants13111570/s1, Figure S1: The chromatogram of UPLC.
