# Supplementary figures and images for: Identification and Functional Characterization of the SaMYB113 Gene in Solanum aculeatissimum
Source: Plants (Basel). 2024 Jun 6;13(11):1570. doi: 10.3390/plants13111570 (PMC11174649; doi:10.3390/plants13111570)

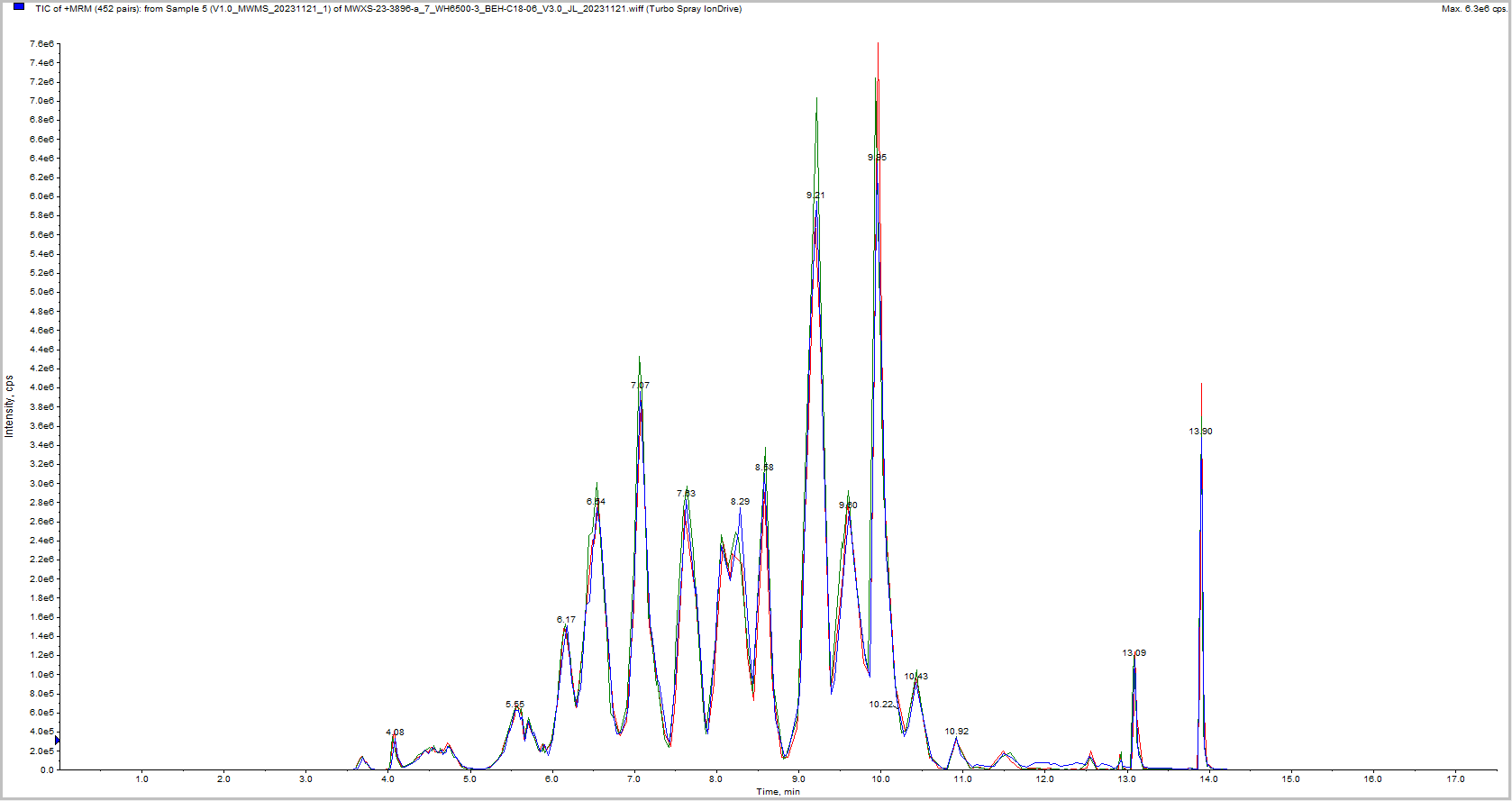

Supplement: Supplementary file 1 [file plants-13-01570-s001.zip › Figure S1. The chromatogram of UPLC.png]
